# Relationship between middle school students’ academic stress and physical exercise behavior from the perspective of Self-Determination Theory: The chained mediation of motivation and intention

**DOI:** 10.1371/journal.pone.0316599

**Published:** 2025-01-03

**Authors:** Kegang Zhao, Yichen Zhao, Weiqiang Xu

**Affiliations:** 1 School of Physical Education, Shandong Normal University, Jinan, Shandong, China; 2 Department of Physical Education, Xiamen University, Xiamen, Fujian, China; 3 Gdansk University of Physical Education and Sport, Gdansk, Poland; University of Tartu, ESTONIA

## Abstract

Academic stress is associated with lower engagement in healthy behaviors, including physical exercise, among middle school students. Based on Self-Determination Theory, this study examines the association between academic stress and physical exercise behavior among middle school students, exploring the mechanisms through the chained mediation of motivation and intention. Scales used in this study include the Academic Stress Scale, Autonomous and Controlled Motivation Scales, and Physical Exercise Intention and Behavior Scales to measure relevant variables. This cross-sectional study involve 290 middle school students (116 males, age = 13.76±0.96 years, grades 7–9) selected from a middle school in Xiamen, China. Structural equation modeling is used to analyze the data, revealing the following results: (1) Academic stress is significantly associated with middle school students’ exercise behavior through the mediating role of exercise intention; (2) Controlled motivation, autonomous motivation, and exercise intention serve as chained mediators between academic stress and exercise behavior; (3) Academic stress is not associated with exercise intention through the parallel mediation of controlled and autonomous motivations. These findings provide new insights into the relationship between academic stress and physical exercise behavior in middle school students.

## 1 Introduction

Physical exercise is crucial for maintaining health. The factors that influence physical exercise have long intrigued researchers from various disciplines. Physical exercise, defined as goal-oriented, planned, and repetitive activity, aims to maintain or improve physical fitness [1]. The middle school years are particularly pivotal, as they are a period of rapidly increasing physical capabilities and the development of exercise habits that can impact long-term health [2, 3]. However, research has shown that adolescents in many countries display low physical activity levels [4–8], excessive sedentary behavior [9], and rising obesity rates [10], significantly challenging their physical and mental health development. Adolescents generally lack adequate physical exercise, which coincides with an increasing obesity rate [11, 12].

Globally, about 80% of adolescents aged 13–15 fail to meet the recommended guidelines [13] of at least 60 minutes of moderate to vigorous daily physical activity, including vigorous activity on at least three days per week. Surveys indicate that American adolescents, especially those aged 9 to 15, participate in physical activities less frequently than public health recommendations suggest, with a notable decrease in activity levels [14, 15]. The 2019 eighth National Physical Fitness and Health Survey of Chinese Students reported that 23.8% of students aged 6 to 22 years met physical fitness and health standards, with only 17.7% of those aged 13 to 22 years doing so. A survey across eight Chinese cities revealed that just 21.8% of adolescents engage in at least one hour of daily physical activity in schools, excluding physical education classes. Outside school hours, 15.9% of adolescents did not exercise from Monday to Friday, 35.0% exercised for less than 30 minutes, 13.2% never exercised on weekends or holidays, and 34.0% also exercised for less than 30 minutes [16].

Research indicates economic constraints, school sports policies, and inadequate sports facilities primarily limit middle school students’ participation in physical exercise. However, academic stress is a major barrier to their participation in physical activities [16, 17]. In China, an overemphasis on academic performance significantly hinders middle school students’ physical activity [18, 19]. Academic stress, stemming from learning-related stimuli, manifests as psychological burden and tension in students. Moderate academic stress can improve cognitive abilities and learning efficiency in students. However, excessive academic stress may lead to adverse emotional states such as fatigue and anxiety in students [20]. Individuals who consistently participate in physical exercise experience significantly lower stress levels than those who do not [21, 22]. Despite extensive research, there remains a critical gap in understanding how academic stress influences physical exercise behaviors among adolescents. Although prior studies have explored factors such as economic constraints, school policies, and inadequate sports facilities, the intrinsic link mechanisms between academic stress and physical exercise behavior have not been sufficiently investigated. This gap is particularly significant given the overemphasis on academic performance in many countries, including China, where academic stress is a major barrier to physical activity.

Many social psychological theories confirm that motivation and intention significantly predict various health behaviors [23, 24]. Self-Determination Theory (SDT) is widely recognized as an effective framework for understanding variations in health-related behaviors and serves as a foundation for designing interventions. Beyond health contexts, SDT has been applied across diverse fields, including internet banking, where it aids in understanding continuance intention by examining motivational factors [25, 26]. Additionally, SDT is extensively utilized in education to clarify the development of motivation and its impact on academic achievement [27, 28]. Comprising theories of basic psychological needs, cognitive evaluation, organismic integration, and causality orientation, SDT offers a comprehensive view of motivational processes. Its strength is in dynamically observing various motivations, especially in terms of continuity and gradation along the autonomy dimension [29]. The early stages of SDT focused on intrinsic and extrinsic motivation, including external regulation, internal regulation, identity regulation, and integrative regulation [30–32]. Theory suggests that autonomous and controlled motivation are inversely related [33, 34]. Later, SDT rejected the dichotomy of intrinsic and extrinsic motivation [35], and emphasized the distinction between autonomous and controlled motivation [36]. Its argues that individuals can be motivated from external sources but can also feel autonomous. Autonomous and controlled motivation are core concepts in SDT today.

**Fig 1:**
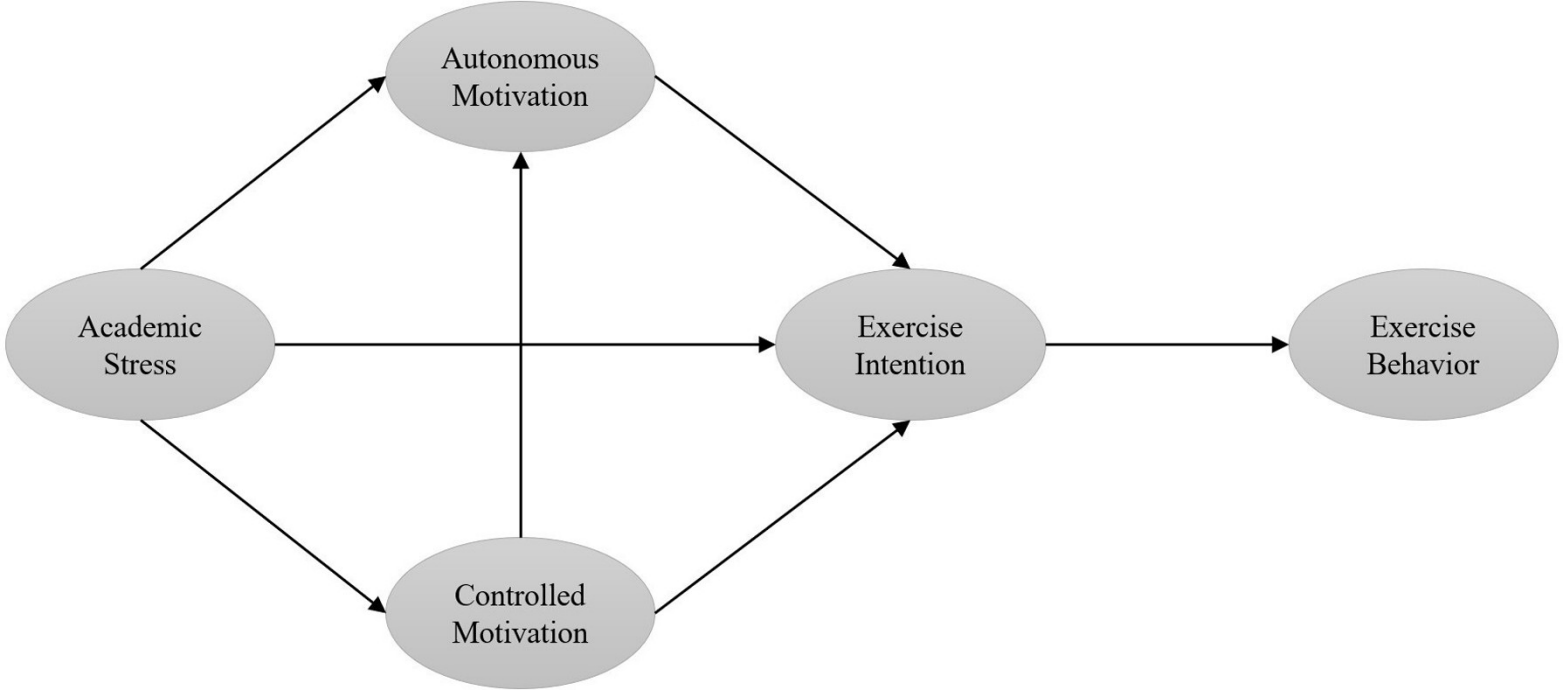
Schematic diagram of the research framework.

In summary, SDT offers a comprehensive framework for exploring motivation development in education and analyzing the link between academic stress and physical exercise behavior. Although recognized as beneficial for adolescents’ physical and mental health, participation in physical exercise remains below recommended levels. Particularly, in the context of increasing academic stress, the physical exercise behavior of middle school students is significantly affected. Prior research has shown some deficiencies in exploring the intrinsic link mechanisms between academic stress and physical exercise behavior. Consequently, this study will apply the SDT framework, particularly focusing on autonomous and controlled motivations, to analyze how motivation and intention mediate the relationship between academic stress and physical exercise behavior. This study aims to examine the association between academic stress and middle school students’ exercise behavior and to identify underlying psychological mechanisms to address gaps in current research. The study’s innovation lies in unveiling the association between academic stress and physical exercise and exploring motivation’s mediating role. It provides new theoretical insights and practical approaches to enhance adolescent health. Fig 1 shows a schematic of the research framework.

## 2 Research hypotheses

### 2.1 The mediating role of exercise intention

Academic stress is the discomfort and mental burden students face while adapting to the learning environment [37], often arising from pressures from teachers, parents, and social interactions [20]. It manifests in measurable changes in students’ physiological, psychological, and social behaviors. Driven by exam-oriented education, schools place excessive emphasis on academic performance and college admission rates, often neglecting students’ physical health. This results in heavy academic workloads and significant stress for students, leaving them with little energy for physical exercise [38]. Studies show that students who exercise irregularly report higher stress levels than those who exercise regularly [21]. Regular physical exercise is linked to reduced academic stress and can effectively alleviate stress [22, 39]. Higher academic stress is a major barrier to students’ participation in extracurricular physical activities [40]. Behavioral intention reflects an individual’s perceived likelihood and willingness to engage in a specific behavior. Behavior is the actual action undertaken by an individual. All factors that influence behavior do so indirectly through behavioral intention [41]. Therefore, academic stress is associated with students’ exercise behavior indirectly through exercise intention. Based on the aforementioned discussion, the following hypothesis is proposed:

H1: Academic stress is significantly associated with exercise behavior through the mediating role of exercise intention; higher stress is associated with lower exercise intention and reduced exercise behavior.

### 2.2 Parallel mediation of controlled and autonomous motivations

Research indicates that within SDT, various motivations are significantly associated with athletes’ behavioral intentions [42, 43], activity persistence [44, 45], and performance [46, 47]. Controlled motivation is negatively correlated with performance, in contrast to autonomous motivation [48, 49]. A meta-analysis by Katherine of 46 studies revealed a moderate positive correlation between autonomous motivation and physical exercise, and a weaker negative correlation for controlled motivation [50]. In China, middle school students are required to take a physical education entrance exam for high school that assesses various athletic skills via a standardized test. Since physical education comprises 5% to 10% of the total entrance exam score, students are pressured to enhance their performance in this area.

According to SDT, controlled motivation occurs when external factors like others’ expectations and rewards/punishments influence individuals’ behaviors, driving them through pressure and obligation. Pressure to excel in physical education enhances students’ controlled motivation to engage in physical exercise [30]. Overly high expectations from teachers and parents increase students’ academic stress, which may lead to perceived reduced support and hinder the internalization of external motivations, weakening their autonomous motivation [30]. Studies indicate that increased autonomous motivation is associated with positive behavioral intentions, whereas increased controlled motivation is associated with negative behavioral intentions [51]. Consequently, academic stress is indirectly associated with exercise intention through both controlled and autonomous motivations. Based on the aforementioned discussion, the following hypotheses are proposed:

H2: Higher academic stress is negatively correlated with autonomous motivation, which in turn is positively correlated with exercise intention.
H3: Higher academic stress is positively correlated with controlled motivation, which is negatively correlated with exercise intention.

### 2.3 Chain mediation of controlled motivation, autonomous motivation, and exercise intention

Although SDT traditionally views autonomous and controlled motivations as opposite extremes on a continuum, recent research tends to regard these motivations as independent continua, which is considered more accurate both theoretically and empirically [52]. Taking Staw [53] noted that although economic incentives, promotion opportunities, and supervisory expectations are common in organizations, leading to frequent experiences of controlled motivation among employees, they may also discover intrinsic interests in their work, thereby stimulating autonomous motivation. Research by Amabile et al. [54] confirms this, showing in college students and working adults that autonomous and controlled motivations are independent and essentially orthogonal.

Therefore, an individual’s actions may arise from personal interest, external pressure, a combination of both, or neither. When individuals take proactive actions, they employ self-control, which is considered a finite psychological resource. Thus, using self-control can temporarily deplete this psychological resource [55]. Controlled motivation-driven proactive actions increase psychological resource consumption due to pressure, potentially reducing autonomous motivation [56]. The crowding-out effect of motivation shows that external incentives shift the behavioral drive from internal desires to external pressures, displacing internal with external motivation [57]. This suggests that external incentives, as a form of controlled motivation, could weaken autonomous motivation. Therefore, higher levels of controlled motivation may be associated with lower levels of autonomous motivation. Based on the aforementioned discussion, the following hypothesis is proposed:

H4: Academic stress is associated with higher controlled motivation and lower autonomous motivation and exercise intention, which together may be associated with reduced exercise behavior.

## 3 Materials and methods

### 3.1 Procedures

This study adopted a positivist paradigm to explore the relationship between academic stress and exercise behavior through quantitative data analysis. Following a cross-sectional design, the study focused on middle school students in Xiamen, China. Participants were recruited with the assistance of school staff, and data were collected in a classroom setting to maintain consistency. A simple random sampling method was used to select 400 students from over 1,000 eligible participants, with 392 completing the questionnaire. After excluding 102 incomplete responses or non-serious questionnaires [58, 59], 290 valid responses were retained, resulting in a response rate of 72.5%. This approach provided each student in the target population an equal chance of selection, enhancing sample representativeness and strengthening the external validity of the findings. Data collection occurred from June to July 2024.

Inclusion criteria required participants to be male or female students between 12 and 15 years old, currently enrolled in grades 7 to 9 in a middle school in Xiamen, China, with informed consent from both participants and guardians. Exclusion criteria included students outside the specified age or grade range, those with medical conditions limiting physical exercise, those previously participating in similar studies, and those who did not provide informed consent.

The study adhered to the principles of the Declaration of Helsinki. Ethical approval was obtained from the Ethics Committee, School of Physical Education, Shandong Normal University (Approval Number: SDNUTYDW2024010), ensuring all procedures met ethical guidelines. Written informed consent was obtained from all participants and their legal guardians prior to the survey, and participation was entirely voluntary.

### 3.2 Participants

The final sample included 290 students, with an average age of 13.76 ± 0.96 years. Details on gender distribution, grade levels, and physical exercise frequency over the past two weeks are presented in Table 1. The minimum required sample size was calculated using R software, based on 112 degrees of freedom. The minimum sample size was 166, while the effective sample size of this study was 290, providing a statistical power of 0.992, which surpasses the recommended threshold of 0.8 and indicates robust power.

**Table 1:** Characteristics of study participants.

Characteristic	Category	Count	Percentage
**Gender**	Male	116	40%
	Female	174	60%
**Age** (13.76 ± 0.96)	12	28	9.7%
	13	92	31.7%
	14	92	31.7%
	15	78	26.9%
**Grade level**	First-year (Grade 7)	112	38.6%
	Second-year (Grade 8)	53	18.3%
	Third-year (Grade 9)	125	43.1%
**Physical Exercise Frequency in the past two weeks**	0 times	11	3.8%
	1 time	11	3.8%
	2 times	35	12.1%
	3 times	60	20.7%
	4 times	52	17.9%
	5 times	39	13.4%
	6 times	82	28.3%

### 3.3 Measures

#### 3.3.1 Academic stress

Adapted from Xu Jiajun’s [20] academic stress scale, the original version included four dimensions: self-imposed pressure, teacher pressure, social pressure, and parental pressure. Due to its lower consistency with the other dimensions, self-imposed pressure was removed from the revised scale, which now includes only teacher, social, and parental pressures across 13 items on a 7-point Likert scale. The teacher pressure scale includes items like “The teacher will criticize me when I cannot answer their questions.” The social pressure scale covers scenarios such as “I feel I lack a close friend to talk to when troubled,” and the parental pressure scale includes “My parents get angry when I perform poorly on exams.” This study used principal component analysis with varimax rotation for exploratory factor analysis to validate the scales. Internal consistency was measured using Cronbach’s alpha. Reliability analysis yielded the following results: Cronbach’s alpha for the teacher pressure scale was 0.731, for the social pressure scale 0.715, and for the parental pressure scale 0.816. After simplifying second-order factors to first-order, the academic stress scale’s Cronbach’s alpha reached 0.666, indicating reliable results for middle school samples.

#### 3.3.2 Autonomous and controlled motivations

Scales for autonomous and controlled motivations, adapted from research by Chan D.K., Battistelli A., Standage M., et al. [60–62], utilize selected items on a 7-point Likert scale. The autonomous motivation scale includes 5 items, such as “I participate in physical exercise because I enjoy it” and “Engaging in physical exercise is part of my life”. The controlled motivation scale contains 4 items, such as “I participate in physical exercise but I question why I should” and “I engage in physical exercise because I want my teacher to think I am a good student”. Reliability assessment results: Cronbach’s alpha was 0.950 for the autonomous motivation scale and 0.692 for the controlled motivation scale. This demonstrates good reliability for both scales, confirming their suitability for middle school samples.

#### 3.3.3 Exercise intention and exercise behavior

The exercise intention scale in this study, adapted from the Physical Activity Planned Behavior Questionnaire by González et al. [63], uses a 7-point Likert scale. The scale includes 3 items, such as “I have considered engaging in physical exercise at least 6 times in the next two weeks”. The exercise behavior scale, based on the design by Zhang Qiang [64], also uses a 7-point Likert scale. This scale includes 2 items: “The number of times I have engaged in physical exercise in the past two weeks” and “The average daily duration of physical exercise I have engaged in over the past two weeks”. The physical exercise investigated in the questionnaire refers to extracurricular physical exercise, excluding school physical education classes. Reliability assessment results were: Cronbach’s alpha of 0.897 for the exercise intention scale and 0.632 for the exercise behavior scale. The findings confirm that both scales have good reliability, making them suitable for middle school samples.

### 3.4 Data analysis

This study used SPSS 26.0 for descriptive statistics and correlation analysis, and AMOS 26.0 to assess the scales’ reliability and validity. Common method bias was assessed using Confirmatory Factor Analysis (CFA), and hypotheses were tested via Structural Equation Modeling (SEM). Additionally, the study employed the Bootstrap resampling method, using 5000 samples, to examine both parallel and chained mediating effects among variables at a 95% confidence interval [65, 66]. SEM was chosen for its ability to simultaneously process multiple dependent and independent variables, crucial for analyzing complex interactions in our research. SEM is particularly useful for mediation analysis, offering insights into the associations between academic stress and exercise behavior through the mediating roles of motivation and exercise intention. SEM analysis involved evaluating statistical power, with higher values indicating more accurate research results. Even with a good model fit, lower statistical power could compromise result accuracy [67].

## 4 Results

### 4.1 Common method variance analysis

This study used confirmatory factor analysis (CFA) to assess common method variance (CMV). The presence of CMV was assessed by evaluating a significant increase in the chi-square value of a single-factor CFA model [68]. Results indicated that the chi-square value was 1004.898 (df = 119) for the single-factor CFA model and 235.140 (df = 109) for the multifactor CFA model. The chi-square difference Δχ2 = 769.758 (Δdf = 10) was significant, with STATBLW software calculations yielding p<0.001. Consequently, the absence of CMV between the two models enhances the reliability of the results.

### 4.2 Confirmatory factor analysis

Confirmatory factor analysis (CFA), essential to SEM, verifies the measurement model’s accuracy to ensure it accurately represents the research’s key dimensions. The analysis utilized AMOS 26.0 software. Composite reliability (CR) reflects the internal consistency of latent variables, serving as a comprehensive reliability indicator. In this study, all CR values surpassed 0.6, demonstrating good internal consistency of the constructs. Average Variance Extracted (AVE) quantifies the explanatory power of latent variables, where higher values suggest stronger reliability and convergent validity. Ideally, AVE values should exceed 0.5. However, if CR values are above 0.6, AVE values below 0.5 may still represent adequate convergent validity. AVE values ranging from 0.36 to 0.5 are considered acceptable [69]. Table 2 shows that factor loadings for all constructs were greater than 0.45, ranging from 0.469 to 0.991, meeting significance criteria. CR values ranged from 0.675 to 0.951, demonstrating strong internal consistency. The AVE values were 0.794 for autonomous motivation and 0.758 for exercise intention, both surpassing the standard. Although AVE values for academic stress and controlled motivation fell below 0.5, CR values above 0.6 and AVE within the 0.36 to 0.5 range suggest effective convergent validity.

**Table 2:** Confirmatory factor analysis.

Latent Variables	Observational Variables	Significance Test Parameters				Std.	SMC	CR	AVE
		Unstd.	S.E.	Z-value	P				
**Academic Stress**	TP^a^	1.000				0.528	0.279	0.675	0.416
	SP^b^	1.605	0.283	5.679	p < 0.001	0.782	0.612		
	PP^c^	1.286	0.202	6.364	p < 0.001	0.599	0.359		
**Autonomous Motivation**	AM1^d^	1.000				0.823	0.677	0.951	0.794
	AM2	0.941	0.057	16.575	p < 0.001	0.808	0.653		
	AM3	1.112	0.052	21.545	p < 0.001	0.947	0.897		
	AM4	1.090	0.052	20.984	p < 0.001	0.933	0.870		
	AM5	1.110	0.053	21.052	p < 0.001	0.935	0.874		
**Exercise Intention**	EI1^e^	1.000				0.796	0.634	0.903	0.758
	EI2	1.167	0.063	18.395	p < 0.001	0.977	0.955		
	EI3	1.023	0.063	16.359	p < 0.001	0.829	0.687		
**Controlled Motivation**	CM1^f^	1.000				0.605	0.366	0.692	0.361
	CM2	0.829	0.134	6.186	p < 0.001	0.524	0.275		
	CM3	1.043	0.155	6.713	p < 0.001	0.609	0.371		
	CM4	1.182	0.172	6.858	p < 0.001	0.659	0.434		
**Exercise Behavior**	EB1^g^	1.000				0.991	0.982	0.728	0.601
	EB2	0.425	0.103	4.130	p < 0.001	0.469	0.220		

^a^Pressure from teachers.

^b^Social Pressure.

^c^Pressure from parents.

^d^Autonomous motivation.

^e^Exercise intention.

^f^Controlled motivation.

^g^Exercise behavior.

### 4.3 Discriminant validity

This study employed the Average Variance Extracted (AVE) method to assess discriminant validity. According to Fornell and Larcker [69], determining discriminant validity requires considering both convergent validity and the correlation between constructs, demanding that the square root of each construct’s AVE exceeds the correlation coefficients among constructs. As Table 3 shows, the square roots of all constructs’ AVE exceed the standardized correlation coefficients of the remaining constructs, thereby demonstrating that the model possesses discriminant validity.

**Table 3:** Discriminant validity.

	Exercise Behavior	Controlled Motivation	Exercise Intention	Autonomous Motivation	Academic Stress
**Exercise Behavior**	***0*.*775***				
**Controlled Motivation**	-0.303	***0*.*601***			
**Exercise Intention**	0.488	-0.463	***0*.*871***		
**Autonomous Motivation**	0.280	-0.595	0.536	***0*.*891***	
**Academic Stress**	-0.225	0.587	-0.376	-0.294	***0*.*645***

Bolded numbers in diagonal italics are Average Variance Extracted (AVE) square roots.

### 4.4 Goodness of Fit Index

In Structural Equation Modeling (SEM), a sample size over 200 can inflate the chi-square value, thus affecting model fit. Therefore, this study applied the Bollen-Stine Bootstrap method to adjust the fit. The adjusted model fit results are detailed in Table 4, using nine widely recognized model fit indices to report the research findings [70]. All adjusted fit indices met the required standards, confirming the reliability of the research results.

**Table 4:** Discriminant table of Goodness of Fit Index.

Goodness of Fit Index	Allowable Range	Goodness of Fit	Model Fit Discriminations
**Chi-square**		236.811	
**Degree of freedom**		112	
**CFI** ^a^	> 0.90	0.956	Pass
**RMSEA** ^b^	< 0.08	0.062	Pass
**NNFI** ^c^	> 0.90	0.947	Pass
**GFI** ^d^	> 0.90	0.917	Pass
**NFI** ^e^	> 0.90	0.921	Pass
**χ**^**2**^ **/ df**	< 3	2.114	Pass
**AGFI** ^f^	> 0.80	0.886	Pass

^**a**^Comparative Fit Index.

^**b**^Root Mean Square Error of Approximation.

^**c**^Non-Normed Fit Index.

^**d**^Goodness of Fit Index.

^**e**^Normed-Fit Index.

^**f**^Adjusted Goodness of Fit Index.

### 4.5 Hypotheses test

**Fig 2:**
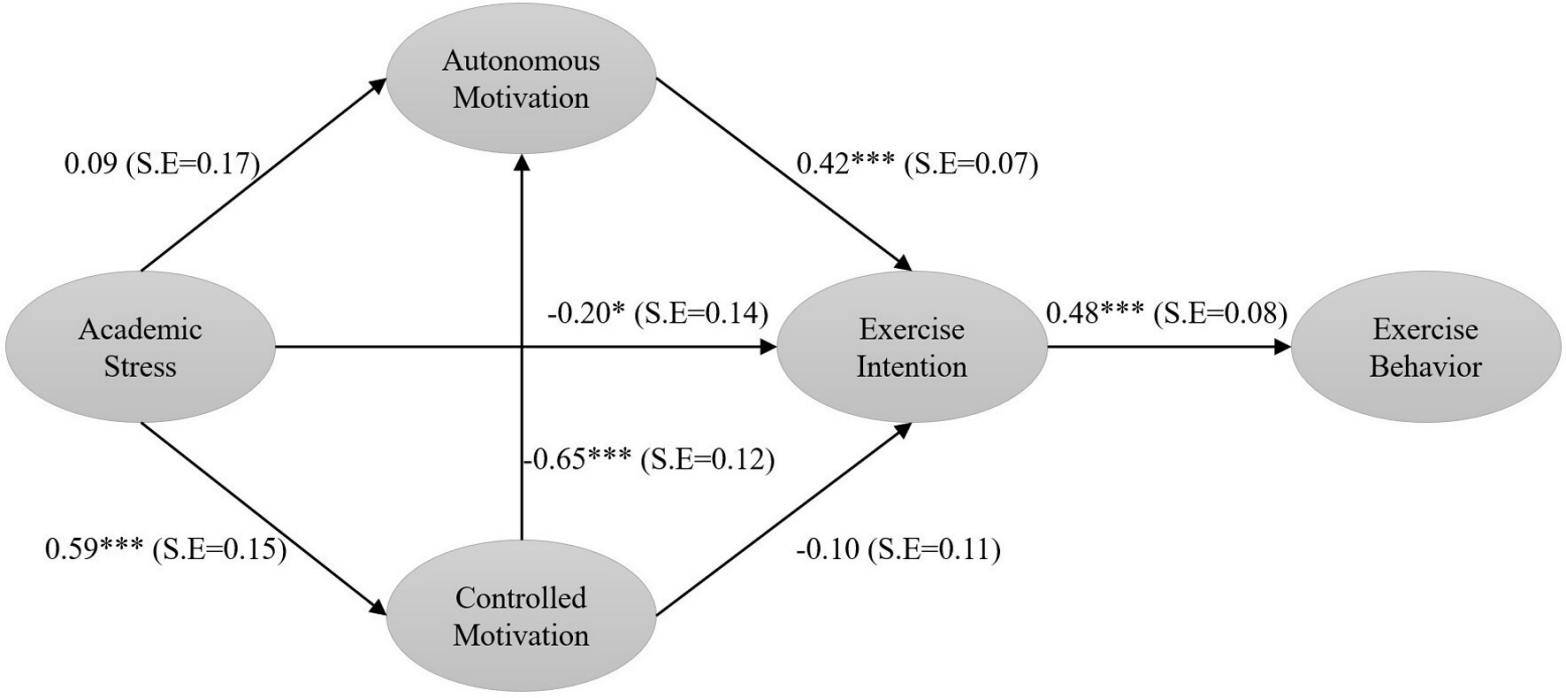
Schematic diagram of the results of the structural equation modeling test.

After confirming each construct’s reliability and validity, this study utilized Structural Equation Modeling (SEM) to test the mediating effects. The Bootstrap method assessed the standard error and calculated the significance levels of the mediating effects. The Bootstrap method provides greater statistical power for testing indirect effects than causal steps and product of coefficients approaches [71, 72]. The Sobel Z Test is frequently used to assess the magnitude of mediating effects [73]. Fig 2 shows a schematic diagram of the results of the structural equation modeling test. Results indicated academic stress was associated with autonomous motivation (γ = 0.15, P>0.05), controlled motivation (γ = 0.91, P<0.05), and exercise intention (γ = -0.31, P<0.05). Controlled motivation was associated with autonomous motivation (γ = -0.71, P<0.05) and exercise intention (γ = -0.10, P>0.05). Autonomous motivation was associated with exercise intention (γ = 0.39, P<0.05), and exercise intention influenced exercise behavior (γ = 0.69, P<0.05). Table 5 shows the significant mediating effect of exercise intention between academic stress and exercise behavior (Z = -1.732, S.E. = 0.123, Bias-Corrected 95%CI [-0.488, -0.012]). The mediating effect of autonomous motivation between academic stress and exercise intention was insignificant (Z = 0.737, S.E. = 0.076, Bias-Corrected 95%CI [-0.062, 0.251]). The mediating effect of controlled motivation between academic stress and exercise intention was also insignificant (Z = -0.714, S.E. = 0.133, Bias-Corrected 95%CI [-0.373, 0.157]). The mediating effects of controlled motivation, autonomous motivation, and exercise intention between academic stress and exercise behavior were significant (Z = -2.582, S.E. = 0.067, Bias-Corrected 95%CI [-0.384, -0.085]). Overall, the total association between academic stress and exercise behavior was significant (Z = -3.664, S.E. = 0.116, Bias-Corrected 95%CI [-0.690, -0.223]). Therefore, hypotheses H1 and H4 were supported, while hypotheses H2 and H3 were not supported.

**Table 5:** Intermediation effectiveness test analysis form.

Effect	Estimate	Product of Coefficients		Bootstrapping			
				Bias-Corrected 95%CI		Percentile 95%CI	
		S.E.	Z	Lower	Upper	Lower	Upper
AS^a^→EI^b^→EB^c^	-0.213	0.123	-1.732	-0.488	-0.012	-0.483	-0.010
AS→AM^d^→EI	0.056	0.076	0.737	-0.062	0.251	-0.084	0.217
AS→CM^e^→EI	-0.095	0.133	-0.714	-0.373	0.157	-0.360	0.176
AS→CM→AM→EI→EB	-0.173	0.067	-2.582	-0.384	-0.085	-0.328	-0.071
Total Effect	-0.425	0.116	-3.664	-0.690	-0.223	-0.675	-0.210

^a^Academic Stress.

^b^Exercise intention.

^c^Exercise behavior.

^d^Autonomous motivation.

^e^Controlled motivation.

## 5 Discussion

This study examined the relationship between academic stress and physical exercise behavior in middle school students, focusing on the mediating roles of motivation and intention. Main findings: (1) Academic stress is negatively associated with exercise intention, which is positively associated with exercise behavior, with exercise intention serving as a mediator; (2) Autonomous motivation does not significantly mediate between academic stress and exercise intention; (3) Controlled motivation does not significantly mediate between academic stress and exercise intention; (4) Academic stress is positively associated with controlled motivation, which is negatively associated with autonomous motivation. Autonomous motivation is positively associated with exercise intention, which is further associated with physical exercise behavior, establishing a chained mediating effect.

### 5.1 Academic stress is indirectly associated with exercise behavior through exercise intention

This study revealed that academic stress is negatively associated with middle school students’ exercise intention, which positively influences their exercise behavior. Initially, research on physical exercise primarily examined its impact on academic stress [74]; however, investigations into how academic stress affects exercise behavior have been limited. Indeed, stress significantly influences physical exercise participation [16, 75], especially among students, where high academic stress (57%) has been identified as a major barrier to participating in extracurricular physical exercise [40]. Theories suggest that high-demand jobs can reduce an individual’s willingness or ability to engage in regular physical activity [76]. This finding was confirmed by a study with 46,573 participants in Finland [77]. Similarly, a long-term Danish cohort study demonstrated that individuals under high stress are twice as likely to engage in less physical activity than those with lower stress levels [78]. Given that “physical exercise” is a specific form of “physical activity”, which is planned, structured, repetitive, and aimed at improving physical health [1], participating in it requires significant mental engagement and can be particularly challenging under stress, which further drains resources.

Behavioral intention, influenced by attitude, subjective norm, and perceived behavioral control, mediates the indirect effects of various factors on behavior [41]. Subjective norm relates to individual decisions made under social pressure. Research shows that if a person’s willpower has been depleted by prior self-discipline, their executive function will be disrupted. Therefore, when they attempt to self-regulate to do what they “should” do, they are less likely to successfully self-regulate to conform to subjective norms [79]. Stress exacerbates the need for self-regulation and weakens executive function, shifting cognitive resources towards uninternalized social expectations. Perceived behavioral control, reflecting facilitators or barriers to action, can directly and indirectly predict behavior through intention. Consequently, if students perceive themselves as less capable of engaging in physical exercise, this perception may reduce their exercise intention and subsequently decrease their participation. Studies indicate that work stress can diminish an individual’s perceived control over physical exercise [80].

The close relationship between stress and the antecedent of behavioral intention suggests that stress can predict behavioral intention. Since behavior is directly influenced by behavioral intention, stress indirectly affects behavior through the mediating role of behavioral intention. Academic stress, originating from external factors such as course load, parental pressure, teacher pressure, and social pressure, can be so overwhelming that it encroaches on leisure time and depletes the energy required for physical exercise, thereby reducing the intention to participate in physical exercise and decreasing physical exercise behavior. Therefore, reducing academic stress, creating supportive environments and opportunities for physical exercise, and boosting exercise intentions are key to enhancing students’ physical exercise levels.

### 5.2 Chain mediation of controlled motivation, autonomous motivation, and exercise intention

Controlled motivation, autonomous motivation, and exercise intention are intermediary variables in the association between academic stress and exercise behavior. Research indicates that external pressure affects adolescents’ motivation for sports, potentially related to changes in perceived causality due to experiencing stress or a sense of control [81]. Perceived causality is the extent to which individuals believe they initiate an action, reflecting the degree of choice they perceive [31, 82]. Autonomous motivation is characterized by a sense of choice, willpower, and independence from external pressures, with individuals high in autonomous motivation tending to act out of self-identification; controlled motivation refers to actions driven by external rewards, demands, or pressures, with individuals high in this type of motivation more likely to act for external reasons. Intrinsic motivation, a type of autonomous motivation, reflects the pursuit of inherent pleasure and satisfaction in activities, independent of external influences. Research shows that intrinsic motivation is an important predictor of continued engagement in physical exercise [36, 83]. Consequently, stress is associated with changes in motivation, which in turn is linked to behavioral intention, positioning controlled motivation, autonomous motivation, and behavioral intention as intermediary variables between stress and behavior.

Controlled motivation is defined as the motivation to engage in a specific behavior under pressure or coercion [84]. This pressure may stem from external factors, such as obtaining rewards or avoiding criticism, where students may participate in physical exercise due to parents’ or teachers’ demands; or from internal factors, like improving self-worth or avoiding shame and guilt, where students may exercise out of a need for self-verification. Faced with physical education entrance exams, students might increase their controlled motivation to participate in physical exercise, spurred by encouragement from parents and teachers. Consequently, academic stress may be positively associated with middle school students’ controlled motivation to engage in physical exercise. Autonomous motivation, characterized by volition and self-recognition, stems from identifying with the values and importance of an action. Through this identification, students may recognize the significance of participating in physical exercise. Autonomous motivation may stem from the pleasure or intrinsic satisfaction derived from engaging in physical exercise. When students experience this intrinsic motivation, they are more likely to enjoy physical exercise. Therefore, autonomous motivation is likely to be positively associated with behavioral intention. Self-control is a finite psychological resource that is depleted during action [55]. This depletion is more pronounced under controlled motivation [56], which in turn impairs autonomous motivation.

According to the motivation crowding effect, external incentives or pressures may shift internal behavioral motivations to external ones, causing external motivations to replace internal motivations [57]. These external incentives or pressures are typically categorized under controlled motivation. For instance, while parental or teacher encouragement may boost students’ controlled motivation for physical exercise, it might simultaneously diminish their autonomous motivation due to the motivation crowding effect. However, this study indicates that controlled motivation may not directly influence exercise intention. This suggests that investigating the relationship between controlled and autonomous motivation through the lens of the motivation crowding effect could provide greater explanatory insight. Specifically, controlled motivation may be negatively associated with autonomous motivation.

### 5.3 Parallel mediation of controlled and autonomous motivations

Academic stress is not effectively associated with exercise intention through the parallel mediation of controlled and autonomous motivations. Additionally, the direct association between academic stress and autonomous motivation is not significant. Previous studies, drawing on the motivation crowding effect, internalization of external motivations, and resource allocation theory, have suggested that stress could influence autonomous motivation [57]. However, this study found that academic stress is not directly associated with autonomous motivation. Autonomous motivation, deriving from an individual’s intrinsic interests and core values, exhibits a degree of resilience. While stress can impact emotional states and cognitive functions, it generally does not alter an individual’s intrinsic interests or values. Therefore, individuals often maintain their intrinsic motivation for physical exercise, even in stressful conditions. Controlled motivation, linked closely to external pressures, often leads individuals to engage in behaviors driven by the need to meet external demands or avoid negative outcomes when under such pressures. This study shows that academic stress may be indirectly associated with autonomous motivation through controlled motivation. While stress may not directly alter autonomous motivation, prolonged or intense stress can lead individuals to feel their choices are constrained, indirectly diminishing their ability to act on intrinsic interests or values. For instance, prolonged academic stress can result in student fatigue and negative emotions, potentially reducing their autonomous motivation for physical exercise. Thus, although academic stress is not directly related to autonomous motivation, academic stress is positively related to controlled motivation, which is negatively related to autonomous motivation.

Controlled motivation is not directly associated with exercise intention. Controlled motivation encompasses both external and intake regulation, with the latter serving as a partially internalized external motivator with higher persistence than purely external motivation. However, intake regulation remains controlled rather than autonomous and is therefore less stable than autonomous motivation, which involves a conflict between external pressure to act and lack of personal intention to act [85]. Controlled motivation is often associated with external rewards, punishments, or other extrinsic pressures that may drive individuals to adopt specific behaviors that may not be fully consistent with the individual’s intrinsic desires or interests. Thus, while controlled motivation may trigger certain behaviors, these behaviors may lack persistence and consistency. For example, research has shown that intake regulation is ineffective for long-term adherence to exercise training [86]. This indicates that controlled motivation has a limited effect on the persistence of physical activity, and its correlation with physical activity is significantly lower than that of autonomous motivation [87]. It has been found that although controlled motivation is primarily driven by external factors, it may still be indirectly associated with exercise intention through the pathway of autonomous motivation. When controlled motivation is integrated with an individual’s intrinsic interests or values, it may enhance the perceived intrinsic value of the behavior and, as a result, indirectly influence behavioral intentions through autonomous motivation. For example, an individual may initially begin to participate in a physical activity due to external rewards, but with practice, he may gradually develop a genuine interest in the activity, which in turn leads to autonomous motivation.

## 6 Limitations

This study presents several limitations. First, the questionnaire, despite being validated for reliability and validity, lacks widespread application. Future studies should use this questionnaire across diverse sample groups to further validate and refine its items. Secondly, as this study relies on self-reported questionnaires, the students’ responses could be subjective. Therefore, caution is advised when interpreting the results of this study. Future research should enhance questionnaire data with observational tracking of middle school students’ physical exercise behaviors to enable data triangulation. Thirdly, as this is a cross-sectional study, it can only confirm the correlations between variables. To verify causal relationships, future research should adopt a longitudinal design. Lastly, while many students participated, all were from the same school in Xiamen, China. Student conditions in other regions or schools may differ, warranting caution in applying these results more broadly. Future research should expand the scope of the survey to include students from diverse backgrounds and regions to enhance the generalizability of the research.

## 7 Practical implications

The findings highlight that academic stress is associated with middle school students’ physical activity, suggesting that managing this stress could improve their physical and mental health. Recognizing academic stress as a barrier point to the need for school-based awareness programs that promote a balance between academics and exercise. Additionally, the study emphasizes the role of autonomous motivation, as students who are self-motivated are more likely to engage in regular exercise. Schools can support this by creating an autonomy-supportive environment, offering activities that align with students’ interests and fostering sustainable health habits.

## 8 Conclusions

This study examines the indirect relationship between academic stress and exercise behavior among middle school students, with particular attention to the mediating roles of controlled motivation, autonomous motivation, and exercise intention. The results show that academic stress is significantly positively correlated with controlled motivation and significantly negatively correlated with exercise intention; controlled motivation is significantly negatively correlated with autonomous motivation; autonomous motivation is significantly positively correlated with exercise intention, which, in turn, is significantly positively correlated with exercise behavior. These findings validate the applicability of Self-Determination Theory (SDT) in explaining the relationship between academic stress and health behaviors among adolescents and expand the application of SDT within the context of academic stress and exercise behavior, offering a new perspective for understanding adolescent health behaviors.

The findings of this study have important implications for educators and policymakers. Reducing academic stress, promoting autonomous motivation, and creating a positive exercise environment can help schools and families effectively enhance students’ exercise participation, thereby improving adolescent physical health. It is particularly important to avoid using external pressure as the primary motivation for exercise, as this may undermine students’ autonomous exercise intentions.

This study is limited by its cross-sectional design, reliance on self-reported data, and a sample restricted to one school in Xiamen, China. Future research should adopt longitudinal designs, include diverse populations, and incorporate observational methods to enhance generalizability and establish causal relationships.

## Supporting information

S1 File Inclusivity in global research. (DOCX)

S2 File (XLSX)
